# Neonatal Sweet syndrome associated with a rectovestibular fistula in a 5-week-old female: A case report

**DOI:** 10.1177/2050313X261463921

**Published:** 2026-07-01

**Authors:** Damy Horth, Isabelle Auger, Marie-Claude Dionne

**Affiliations:** 1 Department of Dermatology, Université Laval, Quebec, QC, Canada

**Keywords:** dermatology, Sweet syndrome, neonatal Sweet syndrome, neutrophilic dermatoses, rectovestibular fistula

## Abstract

Sweet syndrome is an uncommon neutrophilic dermatosis in the pediatric population. As in adults, it is frequently associated with underlying conditions, including infectious diseases, neoplasias, inflammatory bowel disease, and autoimmune or autoinflammatory disorders. Genetic causes, such as chronic atypical neutrophilic dermatosis with lipodystrophy and elevated temperature syndrome, should also be considered, particularly in neonates. We report a case of a 5-week-old female infant with a confirmed Sweet syndrome associated with a rectovestibular fistula—an unusual association, with only three cases previously reported in the literature. To our knowledge, this is the first reported case in a non-Japanese neonate.

## Introduction

Sweet syndrome (SS) is an inflammatory skin condition characterized by the abrupt onset of painful, edematous papules, or plaques. Pustules, vesicules, and ulcerations are frequent. It is usually associated with systemic symptoms, such as fever and eukocytosis as well as elevated inflammatory markers.^1^ The diagnosis is typically confirmed by histopathology, which shows a dense neutrophilic infiltrate in the dermis, sometimes extending into the subcutis, with variable epidermal changes and absence of leukocytoclastic vasculitis. The condition may resolve spontaneously within a few weeks, although recurrences are common.^2^ However, a rapid and dramatic response to systemic corticosteroids is characteristic and constitutes one of the diagnostic criteria.^1^

SS is uncommon in the pediatric population. According to age at onset, it can be subdivided into neonatal (presentation before 3 months of age), infantile (between 3 months and 3 years of age), and juvenile (older than 3 years) SS.^1^ The underlying conditions associated with this neutrophilic dermatosis are similar to those observed in adults and include infectious diseases, neoplasia—most commonly hematologic malignancies, inflammatory bowel diseases, and autoimmune or autoinflammatory disorders.^3^ In neonatal SS, it is particularly important to exclude genetic and immunodeficiency syndromes, such as chronic atypical neutrophilic dermatosis with lipodystrophy and elevated temperature syndrome.^4^

A rare cause of neonatal SS is rectovestibular fistula. Only three cases have been reported in the literature, all in patients of Japanese origin.^5,6,7^ The proposed underlying pathophysiological mechanism involves subclinical infectious and inflammatory responses generated by the fistula, which may be congenital or acquired. This inflammatory milieu leads to cytokine release, promoting neutrophil production in the bone marrow and their migration from the bloodstream into peripheral tissues, including the skin. Combined treatment with systemic corticosteroids and definitive surgical correction of the fistula leads to remission.^5,6,7^

## Case

**Figure 1.**
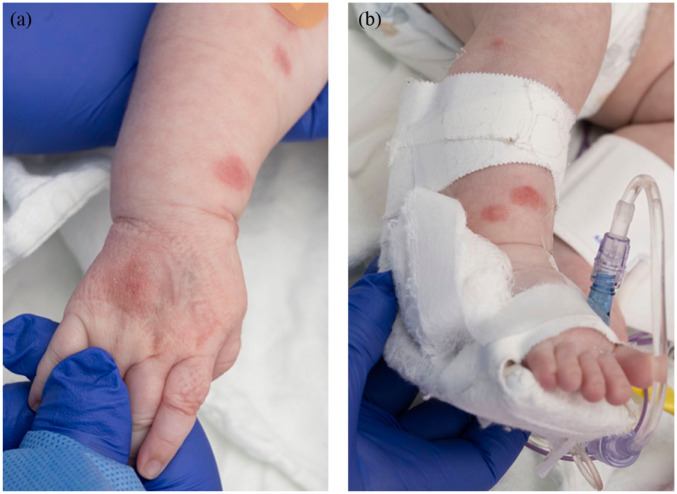
(a) Erythematous and edematous papules and plaques on the right hand and right forearm. (b) Erythematous and edematous papules and plaques on the right pre-tibial area.

A 5-week-old female patient of Maghreb origin was hospitalized in a pediatric hospital in Quebec City in October 2025 for having feced in the vagina. She also presented with erosive lesions on her buttocks and nonspecific papules and pustules on the head and neck. These lesions rapidly evolved into larger papules and plaques with an erythematous and edematous appearance, associated with the development of new lesions on the extremities and ulceration (Figure 1(a) and (b)).

She was afebrile and in good general condition, except for the recent onset of diarrhea. She was born at term and had no significant personal or family medical history. Growth and psychomotor development were within normal limits.

Initial laboratory investigations revealed mild neutrophilia and elevated C-reactive protein levels. Bacterial cultures, as well as polymerase chain reaction testing for herpes simplex virus and varicella-zoster virus, were negative. Given the atypical and rapidly progressive cutaneous presentation, multidisciplinary consultations were obtained, in addition to dermatology, including gastroenterology, immunology, and rheumatology. A thorough physical exam and an extensive workup were unrevealing except for a rectovestibular fistula, with negative investigations for inflammatory bowel disease, primary immunodeficiency, connective tissue diseases, and infectious etiologies.

Two skin biopsies were performed for histopathological examination, as well as bacterial and fungal cultures. Cultures remained negative. Histopathology revealed a dense neutrophilic infiltrate involving the dermis, without evidence of leukocytoclastic vasculitis, consistent with a neutrophilic dermatosis.

Based on the clinical presentation, laboratory findings, and histopathological features, a diagnosis of neonatal SS was made. Given the extensive negative workup and after a thorough review of the literature, the rectovestibular fistula was considered a potential triggering etiology. Three other similar cases have been reported, all occurring in Japanese female pediatric patients.^5,6,7^ Abdominal ultrasonography, electrocardiogram, and transthoracic echocardiography were performed to exclude potential systemic complications of SS and to rule out organomegaly suggestive of an underlying neoplastic process.^1,8^

During the course of hospitalization, the patient required admission to the intensive care unit for acute respiratory distress, clinically suggestive of laryngitis. She was treated with dexamethasone, which was followed by a rapid and marked improvement of the cutaneous lesions. Treatment was subsequently transitioned to oral prednisolone at a dose of 1 mg/kg/day, with a rapid taper over a few days. Complete resolution of the skin lesions was achieved, with no recurrence observed to date. Surgical correction of the rectovestibular fistula is planned by the surgical teams in the coming months.

## Discussion

SS is a rare inflammatory skin disorder classified among the neutrophilic dermatoses. Although uncommon in the pediatric population, its clinical and histopathological features, diagnostic criteria, and underlying causes are similar to those observed in adults. A rare cause of SS in female neonates is rectovestibular fistula. While the exact pathogenesis is not fully understood, immune dysregulation driven by subclinical inflammation and a possible infectious process is thought to play a central role in neutrophil-mediated inflammation and tissue damage. Short-term systemic corticosteroids remain the mainstay of treatment, combined with eventual surgical correction of the fistula. The cases reported in the literature showed a favorable outcome.

In conclusion, this fourth case of neonatal SS associated with rectovestibular fistula highlights the importance of obtaining a thorough history and performing a meticulous physical examination in neonates presenting with SS in order to identify underlying causes. Ongoing follow-up within a multidisciplinary team is recommended to ensure complete resolution.
